# Correction: Meinarovich et al. Diagnosis of Secondary Bacterial Meningitis via Aromatic Metabolites and Biomarkers in Cerebrospinal Fluid. *Int. J. Mol. Sci.* 2025, *26*, 10522

**DOI:** 10.3390/ijms262411856

**Published:** 2025-12-09

**Authors:** Petr A. Meinarovich, Ekaterina A. Sorokina, Natalia V. Beloborodova, Alisa K. Pautova

**Affiliations:** Federal Research and Clinical Center of Intensive Care Medicine and Rehabilitology, Petrovka Str. 25-2, 107031 Moscow, Russia; pmeynarovich@fnkcrr.ru (P.A.M.); esorokina@fnkcrr.ru (E.A.S.); nbeloborodova@fnkcrr.ru (N.V.B.)

In the original publication [1], there was a mistake in the legend for Table 2. The authors mistakenly reported erroneous reference values for 4-hydroxyphenyllactic acid (*p*-HPhLA), 5-hydroxyindole-3-acetic acid (5HIAA), indole-3-lactic acid (3ILA), and indole-3-acetic acid (3IAA).

This has now been corrected. We recalculated all concentrations based on the correct formula: Concentration (ng/mL)/Molar mass (g/mol) * 1000 = Concentration (nmol/L). Table 2 has also been updated accordingly. The correct Table 2 appears below.

Also, there was an error in the original publication [1] in Section 2. Results and Discussion, 2.2. Description of Cerebrospinal Fluid Samples from Cohorts #1 and #2, Paragraph Number 8. This has now been corrected. The correct paragraph appears below.

Concentrations of *p*-HPhLA in patients of all groups generally exceeded the normal values indicated in [26]. Concentrations of 5HIAA and 3ILA in patients without meningitis in groups 1.2 and 2.2 were generally comparable with the norm indicated in [26]. In both groups with meningitis, concentrations exceeding these values are observed. In the case of 3IAA in group 1.2, concentrations comparable to the normal values in [26] were observed.

The authors state that the scientific conclusions are unaffected. This correction was approved by the Academic Editor. The original publication has also been updated.

## Figures and Tables

**Table 2:** The cellular and biochemical composition and concentrations of biomarkers and aromatic metabolites in cerebrospinal fluid samples from patients with sequelae of severe brain damage with a chronic critical illness (cohort #1) and patients after neurosurgical interventions (cohort #2), and the results of comparative statistical analysis (the Wald test) between various groups of samples (statistically significant differences are highlighted in bold).

Parameter	Reference Values	Cohort #1				Cohort #2				*p* _Cohorts #1 and #2_	*p* _1.1 and 2.1_	*p* _1.2 and 2.2_
		All Samples (*n* = 77)	Group 1.1 with Secondary Meningitis (*n* = 21)	Group 1.2 Without Secondary Meningitis (*n* = 56)	*p* _1.1 and 1.2_	All Samples (*n* = 19)	Group 2.1 with Secondary Meningitis (*n* = 12)	Group 2.2 Without Secondary Meningitis (*n* = 7)	*p* _2.1 and 2.2_			
Leucocyte count, cells/mm^3^	2–8	11 (3, 96)	733 (139, 2000)	6 (2, 18)	**<0.001**	163 (18, 710)	258 (136, 866)	14 (5, 88)	0.21	0.27	0.62	**<0.001**
Lymphocytes, %	90–95	61 (30, 94)	11 (5, 36)	75 (54, 100)	**<0.001**	4 (3, 6)	4 (3, 8)	4 (3, 5)	0.55	**<0.001**	0.08	**<0.001**
Neutrophils, %	3–5	47 (25, 68)	84 (69, 92)	40 (22, 50)	**<0.001**	88 (44, 96)	96 (87, 97)	37 (11, 63)	**<0.001**	**<0.001**	0.31	0.57
Protein, g/L	0.1–0.3	0.7 (0.5, 1.2)	1.6 (0.6, 4.5)	0.7 (0.5, 1.0)	**<0.001**	2.6 (1.2, 4.2)	3.6 (2.4, 4.7)	1.2 (0.5, 2.2)	0.06	**<0.001**	0.36	**0.03**
Glucose, mmol/L	2.8–3.9	3.1 (2.2, 3.8)	0.6 (0.2, 3.5)	3.2 (2.6, 3.8)	0.12	2.6 (2.2, 3.5)	2.2 (1.9, 2.7)	3.7 (3.3, 4.2)	**<0.001**	0.42	0.99	0.78
IL-6, pg/mL	1.5 (1.0, 2.2) [23]	71 (10, 897)	3228 (104, 5000)	42 (7, 234)	**<0.001**	2336 (420, 5000)	4017 (1455, 5000)	271 (144, 3371)	0.19	**<0.001**	0.46	**0.03**
NSE, ng/mL	17.3 ± 4.6 [24]	1.6 (0.9, 3.2)	2.0 (1.3, 9.3)	1.3 (0.7, 2.9)	0.06	12.1 (5.0, 37.2)	20.5 (9.0, 35.7)	5.0 (3.3, 64.7)	0.7	0.14	0.4	**<0.001**
S100, μg/L	1.4 ± 0.5 [25]	0.8 (0.4, 3.3)	3.7 (0.7, 18.0)	0.6 (0.3, 1.2)	**<0.001**	6.5 (2.5, 23.1)	5.5 (2.3, 26.0)	9.2 (3.2, 19.6)	0.98	**<0.001**	0.7	**<0.001**
*p*-HPhLA, nmol/L	237 (196, 319) [26]	633 (370, 1246)	2750 (1734, 4206)	491 (281, 840)	**<0.001**	925 (533, 1485)	1172 (861, 1726)	522 (424, 725)	0.46	0.7	0.03	0.1
*p*-HBA, nmol/L	no data	33 (23, 61)	35 (23, 99)	32 (22, 60)	0.26	30 (17, 41)	22 (14, 36)	35 (33, 41)	0.38	0.32	0.31	0.7
*p*-HPhAA, nmol/L	no data	223 (101, 469)	906 (362, 3167)	152 (77, 278)	**0.03**	100 (60, 191)	92 (66, 233)	103 (60, 138)	0.7	0.46	0.32	0.79
PhLA, nmol/L	no data	67 (43, 173)	538 (361, 736)	52 (36, 77)	**<0.001**	100 (59, 131)	108 (94, 145)	47 (35, 68)	0.26	0.57	0.16	0.43
5HIAA, nmol/L	71 (55, 102) [26]	112 (22, 224)	162 (85, 306)	72 (<20, 212)	0.35	122 (81, 204)	143 (103, 215)	72 (62, 146)	0.91	0.87	0.77	0.93
3ILA, nmol/L	9 (7–12) [26]	11 (4, 27)	202 (22, 472)	5 (3, 15)	**<0.001**	25 (18, 85)	60 (25, 93)	10 (6, 18)	0.39	0.95	0.26	**0.03**
3ICA, nmol/L	no data	11 (6, 14)	12 (11, 20)	8 (6, 13)	**0.03**	8 (6, 10)	8 (7, 11)	5 (4, 9)	0.7	0.47	0.17	0.38
3IAA, nmol/L	20 (14–32) [26]	39 (22, 79)	96 (47, 204)	26 (18, 44)	0.12	71 (43, 118)	73 (65, 88)	47 (35, 177)	0.47	0.14	0.6	**0.03**
3IPA, nmol/L	0.11 ± 0.02 [10]	<2 (<2, 4)	2 (<2, 4)	<2 (<2, 4)	**<0.001**	9 (3, 15)	9 (4, 16)	2 (<2, 14)	0.56	0.27	0.88	**<0.001**
